# Physiological and behavioral patterns of corruption

**DOI:** 10.3389/fnbeh.2014.00434

**Published:** 2014-12-22

**Authors:** Tarek Jaber-López, Aurora García-Gallego, Pandelis Perakakis, Nikolaos Georgantzis

**Affiliations:** ^1^Laboratorio de Economía Experimental, Department of Economics, Universitat Jaume ICastellón, Spain; ^2^Department of Economics, University of ReadingReading, UK; ^3^Department of Personality, Evaluation and Psychological Treatment, University of GranadaGranada, Spain; ^4^Economic and Social Sciences Unit, School of Agriculture Policy and Development, University of ReadingReading, UK

**Keywords:** bribery, corruption, skin conductance responses, physioeconomics, auctions

## Abstract

We study the behavior and emotional arousal of the participants in an experimental auction, leading to an asymmetric social dilemma involving an auctioneer and two bidders. An antisocial transfer (*bribe*) which is beneficial for the auctioneer (*official*) is paid, if promised, by the winner of the auction. Some pro-social behavior on both the auctioneers' and the bidders' sides is observed even in the absence of any punishment mechanism (*Baseline*, Treatment 0). However, pro-social behavior is adopted by the vast majority of subjects when the loser of the auction can inspect the transaction between the winner and the auctioneer (*Inspection*, Treatment 1). The inspection and punishment mechanism is such that, if a bribe is (not) revealed, both corrupt agents (the denouncing bidder) lose(s) this period's payoffs. This renders the inspection option unprofitable for the loser and is rarely used, especially toward the end of the session, when pro-social behavior becomes pervasive. Subjects' emotional arousal was obtained through skin conductance responses. Generally speaking, our findings suggest that stronger emotions are associated with decisions deviating from pure monetary reward maximization, rather than with (un)ethical behavior *per se*. In fact, using response times as a measure of the subject's reflection during the decision-making process, we can associate emotional arousal with the conflict between primary or instinctive and secondary or contemplative motivations and, more specifically, with deviations from the subject's pure monetary interest.

## 1. Introduction

A moral dilemma emerges when different motivations of human behavior dictate opposite actions in a given decision-making context. In economic situations, the most appealing type of dilemma concerns the conflict between selfish monetary reward maximization and adherence to some ethical pro-social norm, especially when the latter implies an economic loss. The emotional implications of such conflicts seem to originate, from the interplay between a basic impulse for greedy money-seeking motivations and alternative, more sophisticated social and personal ethical norms^1^. In this paper, we obtain behavioral data and emotional responses by the participants in a laboratory experiment based on a moral dilemma designed as a public procurement auction with the option of an anti-social bribe by the winner of the auction to the auctioneer. Our results indicate that strong emotions are associated with actions against monetary reward maximization, rather than with the fulfillment or the violation of an ethical norm *per se*.

Despite the broadly criticized reductionist construct of an emotionless utility-maximizing machine known as *Homo Oeconomicus*, there is growing consensus among economists that emotions matter in economic decisions. In everyday economic transactions, people act according to intrinsic motivations and ethical standards, often against their pure economic interests. In fact, in many financial decisions, non-economic motivations like a mother's altruism or an activist's ideology, may dominate economic motives. The basic research agenda aimed at explaining human behavior in the presence of conflicting motivations relies on models assuming coexisting alternative objectives in the decision-maker's utility function. Two exceptions to this are Sen's (1977) idea of economically costly commitments to an ethical norm, and a much more recent approach allowing for potentially conflicting multiple selves (Gómez-Miñambres, forthcoming). While both the harmonic and the conflictive approaches can potentially offer explanations for the behavior observed in the presence of incompatible needs and alternatives, it is a major question whether the conflict is perceived as such by the decision-maker or whether it is internalized as a unified problem leading after all to the satisfaction of one's own needs and preferences. The former hypothesis would require accounting for the conflict among opposite attractors of human behavior, potentially leading to emotional arousal.

Several authors have addressed different aspects of corruption in a variety of experimental settings. Regarding the framework adopted, we can distinguish between single-subject^2^ and multi-subject settings. Within the multi-subject paradigm, a variation of which is also adopted in our study, several issues have been addressed, like for example, the role of the matching protocol and the framing of the experimental setup^3^, as well as the role of the subjects' gender and culture^4^ or identity^5^. Many studies seem to focus on mechanisms and institutions which could enhance or mitigate corruption^6^. Also related to our second treatment, Abbink et al. (2014) report a bribery experiment run in India, including a whistle-blowing option. They find that immunity for bribe givers reduces the propensity of bribe-takers to demand bribes and increases the willingness to report, which is found to depend on intrinsic motivations. Finally, a setup which has some similarities with our setting is used by Gneezy et al. (2013), assuming a game involving two workers and a referee, finding that when the referee keeps both workers' bribes, the judgment seems to be more motivated by the workers' real performance. Despite similarities with other settings, our framework is novel and yields interesting insights on the emotional and behavioral aspects of corruption.

The literature on *integral*^7^ emotions associated with specific economic decisions has focused both on the conflicts preceding the decision and on the feelings triggered by feedback received after a decision is made. The existence of emotional arousal during economic decision-making has been confirmed by several studies^8^. In fact, physiological measures of emotion, mostly skin conductance responses, have demonstrated that decision-making may influence and be influenced by somatic markers activated in bioregulatory processes^9^. We contribute to this literature using skin conductance reactions to specific decisions and feedback from them. The framework proposed involves a moral dilemma emerging in the presence of competing motivations resulting from the contrast between selfish monetary reward maximization and pro-social attractors of individual action. Related to our approach, Coricelli et al. (2010) and Coricelli et al. (2014) have established that economic decisions involving some degree of conflict with ethics cause significant emotional and, eventually, somatic reactions. The main conclusion seems to be that violations of specific pro-social norms is reflected on higher levels of arousal, which was shown to relate to the emergence of negative self-reported emotions. Our research extends this view by being the first to show that high emotional arousal may also relate to ethical behavior when a specific decision is made against the purely selfish motivation of monetary reward maximization.

## 2. Material and methods

### 2.1. Theoretical framework

The framework studied here is inspired by Beck and Maher (1986), Lien (1986), Burguet and Che (2004), Che (1993), and, especially, Büchner et al. (2008). We explicitly introduce a tradeoff between bribe and quality bids. In the bidding stage, two firms post-simultaneously sealed quality bids and bribes to be paid in case the bidder is the winner of the auction. In the final stage, on the basis of the bids received, an official chooses one of the bids. The winner's quality benefits all players, whereas the bribe is antisocial and inefficient, as it is paid at the cost of a lower quality and an extra loss by the bribing winner. Thus, firms face a moral dilemma in the sense that the higher a firm's promised bribe, the more likely for the firm to be the winner of the auction. Also, officials face a dilemma, as their selfish preference for bids entailing higher bribes goes against the interest of all other players and overall welfare.

In the experiment, we implement the following payoffs:

(1) $$\pi_{official}\;=F+a\cdot Q_{winner}+B_{winner}$$

(2) $$\pi_{winner}\;=F+a\cdot Q_{winner}-c\cdot B_{winner}+R$$

(3) $$\pi_{loser}\;=F+a\cdot Q_{winner}$$

where *F* is a fixed amount earned by each subject in each period, *Q* and *B* are, respectively, the quality and bribe bids. *R* is the extra monetary reward earned by the winner of the auction. Finally, *a* denotes the social return of the winning project's quality on each player's utility and *c* the cost per monetary unit of bribe tranferred by the winner to the official. In order to limit the actions of firm-subjects to pressing a single button, we have applied the restriction *Q* + *B* = *A* for each firm-subject's strategies, reflecting the trade-off between quality and bribes. We have used the parameter set (*F*, *a*, *c*, *A*, *R*) = (10, ½, 2, 10, 10)^10^.

#### 2.1.1. Monetary payoff equilibrium prediction

Taking this payoff structure into account, implying that agents care only for the monetary consequences of their actions and assuming a continuous strategy space, the unique Nash equilibrium is such that both firms' bids involve (*Q*, *B*) = (5, 5). That is, like in Bertrand competition, firms will be willing to spend on the bribe as much as the bonus they obtain from winning the auction. However, as usual, our experiment is run with a discrete strategy space, allowing only for integer quality and bribe bids. Then, multiple equilibria^11^ emerge including (*Q*, *B*) = (7, 3) and (*Q*, *B*) = (6, 4). In this case, the unique continuous-strategy equilibrium (*Q*, *B*) = (5, 5) becomes a weak equilibrium, because each firm is indifferent between this and posting lower bribes, becoming a loser (with a payoff of 12.5 in both cases).

#### 2.1.2. Psychological payoff equilibrium prediction

We generalize the monetary payoff structure of the setup in (1–3), using a linear specification of utilities with an agent-specific psychological cost parameter, γ, capturing an agent's aversion to bribe due to ethical reasons, expressed as a loss per monetary unit of bribe received by the official. Thus, the three agents' utility levels after the end of the auction are given by:

(4) $$\pi_{official}\;=F+a\cdot Q_{winner}+(1-\gamma_{official})\cdot B_{winner}$$

(5) $$\pi_{winner}\;=F+a\cdot Q_{winner}-(c+\gamma_{winner})\cdot B_{winner}+R$$

(6) $$\pi_{loser}\;=F+a\cdot Q_{winner}$$

Assuming perfect information on the agents' preferences and symmetry in the sense that each firm correctly predicts that its rival has a similar attitude to ethics, the following cases emerge:

1. If *a* ≥ 1 − γ_*official*_, the highest quality project will be chosen by the official and firms will bid only in qualities, leading to the equilibrium: (*Q*, *B*) = (*A*, 0) independently of the firms' preferences.
2. If *a* < 1 − γ_*official*_, the highest bribe will be preferred by the auctioneer. In that case, firms will bid with the maximum bribe they can, as long as the generalized bribing cost does not exceed the fixed amount *R* earned by the winner. Thus, in equilibrium, firm *i* will bid (*Q*_*i*_, *B*_*i*_) = (*A* − $\frac{R}{c+\gamma_{i}}$, $\frac{R}{c+\gamma_{i}}$)^12^.

Summarizing, the model predicts that officials may choose the highest quality proposal if they are sufficiently bribery-averse, while they will choose the bidder with the highest bribe otherwise. In the perfect information setting discussed above, firms faced with a quality-maximizing auctioneer, will not bid with bribes, independently of their own preferences, whereas firms anticipating a bribery-maximizing behavior by the auctioneer will promise higher bribes, the less bribery-averse they are. In the case of uncertainty regarding the official's type, a generalized version of this model would produce a continuum of equilibrium predictions, depending on the percentage of pro-social officials and the distribution of bribery-aversion costs. While the development of a general model with these characteristics is beyond the scope of this paper, it is rather straightforward consequence of our setup that the distribution of officials' and firms' bribery-aversion parameters will have the expected result of less bribery and more pro-social project choices, the higher the density of bribery-aversion parameters on larger values.

### 2.2. Experimental design

Two treatments were run^13^, using a within-subject design. Subjects were aware of the fact that new, supplementary instructions would be given after period 15. During the first 15 periods, the Baseline (T0) treatment was run, corresponding to the auction described above, played repeatedly by fixed triplets of players, each representing an economy of two firms and an official. At the beginning of period 16, the Inspection (T1) treatment was introduced with further instructions and new fixed roles and matching of players. In T1, after each auction has been resolved by the auctioneer, the loser can activate the “Inspect” option to reveal a possible bribe. A revealed bribe leads both players involved to the loss of their period earnings, whereas, if no bribe is revealed, the denouncing firm loses all its period profits instead. This extreme setup for the inspection and punishment mechanism does not affect the theoretical equilibrium predictions of the model, given that, in theory, losers should not use the inspection option because of its negative expected payoff. Given the lower complexity of T0 compared to T1, we kept the order of the two conditions fixed to guarantee that subjects' learning in T0 helped them adapt faster to the more complex situation in T1. This also helped us to avoid having too few observations in any of the subcases (like for example no bribe in T0).

### 2.3. Procedures

A total of 93 subjects participated in the experiment following the usual recruitment and ethical clearance protocols used in the LEE at the Universitat Jaume I (Castellón, Spain)^14^. Given the technical restrictions associated with the continuous measurement of skin conductance, each session consisted of small groups of 12 or 9 subjects each^15^.

#### 2.3.1. Behavioral and physiological data collection procedures

The experiment was computerized using the z-Tree toolbox (Fischbacher, 2007). Continuous electrodermal activity was recorded during the entire experimental session using a BIOPAC MP150 system and four TEL100C telemetry modules (BIOPAC systems, Inc). Two Ag/AgCl electrodes filled with isotonic gel were placed on each subject's distal phalanges of the middle and the index fingers of the non-dominant hand. The BIOPAC amplifier applies a constant voltage of 0.5 V to provide a continuous measure of the skin conductance level between the two electrodes, as this varies with sweat gland activity. Specifically, activation of the sympathetic nervous system due to emotional arousal results in marked increases of the skin conductance level. When evoked by particular experimental events, these increases in conductance are called skin conductance responses (SCRs) (Dawson et al., 2007). The skin conductance signal was sampled at 125 Hz and low-pass filtered offline at 0.5 Hz using a Butterworth digital filter. SCRs were automatically detected and their amplitudes were quantified using a custom version of the Matlab EDA toolbox freely available at: https://github.com/mateusjoffily/EDA. False SCRs were removed after visual inspection of the entire signal. SCRs were associated to a specific decision if their onset appeared at least 1.0 s after subjects were informed about their choices and before the moment of the decision. SCRs were associated to a feedback event when their onset appeared between 1.0 and 3.0 s after the display of the feedback screen. Only responses above 0.02 microSiemens (μS) were considered as valid. Group average SCRs were obtained by averaging across events of the same condition (e.g., decisions involving bribe) the values at each time sample. A non-parametric permutations test based on 200 surrogate data sets, obtained by permutating the data points at each sample from all individual trials of each subject, was subsequently carried out to detect statistical differences between conditions. To control for false positive statistical error, the false discovery rate correction method for multiple comparisons was used.

#### 2.3.2. Timing of behavioral events for continuous-time physiological measurements

Physioeconomics is a new interdisciplinary area of research, combining experimental economics and psychophysiology. Both disciplines use computerized solutions to manage stimuli, strategies, feedback and collect the data. The hardware and software used in our study pose a challenge regarding the need to collect and interactively communicate the timing of behavioral and physiological events across the two computer systems. We have used a solution which to the best of our knowledge has not been previously used in any physioeconomics study so far. As argued in Perakakis et al. (2013), it is the most accurate methodology for the synchronized timing of behavioral data and physiological reactions. The main challenge is how to synchronize, free of undesirable network-related lags, the z-Tree-assisted strategy submission, information screens and feedback on one hand, with the accurate timing (with 2 ms precision) of events necessary for the association of SCR data to their corresponding stimuli. The method relies on the use of photodiodes^16^, detecting the change across subsequent z-Tree screens, on which a small “black box” appears on the upper left corner of each odd-number screen.

## 3. Results

### 3.1. The sample

The behavioral results reported here are based on the sample of 93 participants (31 officials and 62 firm-subjects). Following the two random matchings and role assignments, in T0 there were 15 male officials and 32 male firm-subjects, whereas in T1 there were 13 male officials and 34 male firm-subjects^17^. Physiological results are based on a slightly smaller sample of 89 subjects, given that 4 subjects were excluded due to partly or fully missing information on the recording of their electrodermal activity.

### 3.2. Behavioral results

**Figure 1:**
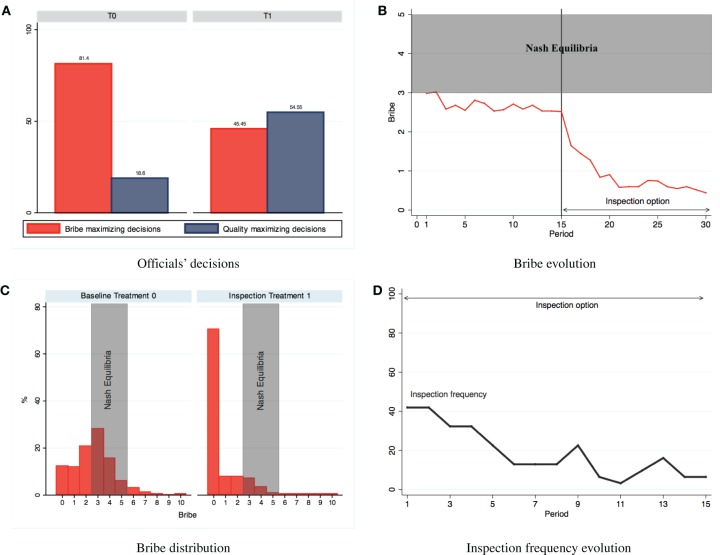
**Behavioral results. (A)** Officials' decisions. **(B)** Bribe evolution. **(C)** Bribe distribution. **(D)** Inspection frequency evolution.

Figure 1 presents the main patterns observed regarding the behavior of subjects. As seen on Figure 1A, in T0, 81.4% of the decisions by officials are compatible with monetary reward maximization (excluding ties, in which the distinction is not meaningful). The remaining decisions (18.6%) are pro-social, corresponding to the choice of the bid with the highest quality, against the official's monetary reward maximization. The pattern is reversed in the presence of an inspection option. Specifically, in T1, the majority of officials' choices (54.55%) become pro-social. Regarding the behavior of firm-subjects in T0, Figure 1B shows a stable pattern of bribe averages slightly below 3 in T0 and a rapidly decreasing trend of bribes in T1. On average, the inclusion of an inspection option in T1 leads to a statistically significant (Mann-Whitney *z* = 15.78, *p* < 0.01) decrease of bribes from 2.66 to 0.81 monetary units. In fact, as seen on Figure 1C, bribe bids in T1 exhibit a high concentration on 0, which becomes the modal strategy chosen by firm-subjects in over a 70% of the cases as opposed to slightly above 10% in T0. Nevertheless, in T0, subjects' behavior has remained within the pro-social range, with almost a fifth of officials' decisions being compatible with a bribery-averse parameter of γ > 1/2 and a bribe average slightly below 3, which is the minimum predicted in the monetary reward-maximizing equilibria. In fact, over 40% of bribe choices are 0, 1, or 2 units. In T1, the introduction of the inspection option has drastically enhanced pro-social behavior, by both officials and firm-subjects, especially toward the last periods, in which average bribes fall below 1, despite the fact that the inspection option has been rarely used (in 87 of 465 or 18.71% of all instances possible) and gradually abandoned by the losers, as shown on Figure 1D, falling from 41.93% (13 out of 31 cases) in period 16 to 6.45% (2 out of 31 cases) in period 30. Interestingly, from all the cases in which an inspection has been activated (*N* = 87), a 44.83% (*N* = 39) corresponds to losing firm-subjects who had offered a bribe in the same period, whereas the majority (55.17% of them, *N* = 48) of inspecting losers had not offered a bribe. In few words, our behavioral results reveal the presence of intrinsic pro-social motivations, but the presence of the extrinsic threat posed by the possibility of an inspection is shown to have a drastic pro-social effect, despite the fact that inspection is only activated in the minority of cases, rendering the threat ex post-efficient, as it enhances pro-social behavior at a negligible social cost.

### 3.3. Physiological results

**Figure 2:**
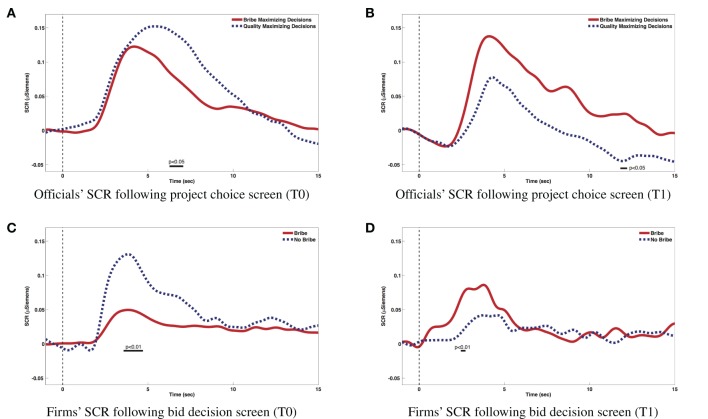
**Emotional responses. (A)** Officials' SCR following project choice screen (T0). **(B)** Officials' SCR following project choice screen (T1). **(C)** Firms' SCR following bid decision screen (T0). **(D)** Firms' SCR following bid decision screen (T1).

From the discussion so far, we have seen that in our setup intrinsic pro-social motivations co-exist with extrinsic motivations like standard monetary reward maximization and the additional threat of punishment for anti-social behavior. Having created these motivations in the laboratory environment, we are now interested in the emotions triggered by different stages of the decision-making process, as well as by the feedback received. Figure 2 displays average SCRs associated to decisions made by officials and firm-subjects. Specifically, Figure 2A shows SCRs related to officials' project assignment decisions in T0. We compare the average SCR related to decisions favorable to bribes with those favorable to a bribe-free bid. We find that decisions deviating from monetary maximization are associated to higher arousal than those giving the license to bribers as dictated by monetary reward maximization (significant differences at *p* < 0.05 were found at a latency range between 6.27 and 7.08 s post-stimulus). Figure 2B shows SCRs corresponding to the officials' project assignment decisions in T1. In this case, decisions opting for the bid with a bribe demonstrate a higher emotional response (*p* < 0.05 between 11.8 and 12.2 s). Before interpreting these findings, we turn to the decisions of firm-subjects. Figure 2C compares the average SCRs associated to decisions made by firms at the moment of posting their bids in T0. Decisions not to bribe entail an increased emotional arousal compared to those who do bribe (*p* < 0.01 between 3.57 and 4.71 s). Finally, Figure 2D represents the same decision event in the inspection treatment (T1), where higher arousal is associated with bribing (*p* < 0.01 between 2.45 and 2.72 s). Therefore, higher arousal levels in T0 are not associated with bribe-giving or bribe-taking but, rather, with individually unprofitable choices. Contrary to T0, in T1, higher arousal levels are associated with bribery. However, observe that while accepting or offering a bribe in T0 is a dominant strategy if we assume that subjects are bribe-neutral and maximize own monetary payoffs, in T1, bribes entail the risk of a significant monetary loss, rendering anti-social behavior individually unprofitable. Thus, a coherent explanation of the 4 patterns observed in Figure 2 is that higher arousal levels correspond to decisions deviating from the objective of maximizing the decision-maker's monetary reward. It is interesting to note that such exciting decisions deviate from the majority choice. In fact, the results obtained for T1 under the threat of being discovered and punished are in accordance with those obtained by Coricelli et al. (2010) and Coricelli et al. (2014), indicating that the negative emotions found in those studies were more likely related with the fear of being discovered to evade than with regret due to non-compliance with a pro-social norm. Therefore, an alternative way to frame or complement the aforementioned explanation is that subjects deviating from pure monetary reward maximization do not only deviate from the strategy dictated by their own pecuniary interest, but also from the strategy chosen by the majority of subjects. An important implication of this finding is that, when choosing in the presence of conflicting motivations, human actions do not equalize (dis)utilities across the alternatives available to them. Instead, conflicts are reflected on emotions which persist after the decision is made and they are perceived stronger, the more a given decision deviates from the basic motivation of monetary reward maximization. Therefore, skin conductance results show an interesting overall pattern concerning bribers. They demonstrate higher arousal when they do not bribe in the baseline treatment and when they do in the inspection one, namely, when they opt for the least common and potentially least profitable strategy in each case. Bribe-takers react in a similar manner, indicating that passive bribery carries also a moral burden.

**Figure 3:**
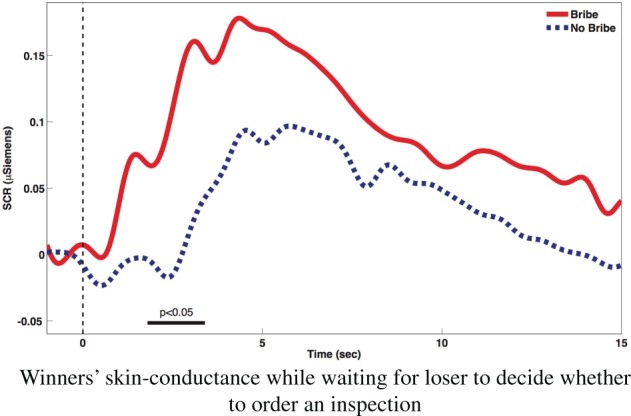
**Winners' skin-conductance while waiting for loser to decide whether to order an inspection**.

While the discussion so far concerns emotions triggered by subjects' decisions, emotional arousal may also emerge from the anticipation of the consequences of others' actions. A rather expected pattern concerns the emotional response obtained due to the anxiety experienced by the winners while waiting for the loser's decision to activate an inspection or not. Figure 3 shows that bribing winners waiting for losers to decide whether to audit them or not exhibit significantly higher emotional arousal compared to honest winners (*p* < 0.05 between 1.8 and 3.4 s).

#### 3.3.1. Response times

**Figure 4:**
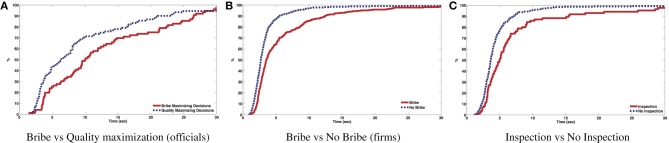
**Response times for T1. (A)** Bribe vs Quality maximization (officials). **(B)** Bribe vs No Bribe (firms). **(C)** Inspection vs No Inspection.

We argue here that emotional arousal emerges from a conflict between monetary and ethical attractors of behavior. Thus, we would expect ethical decisions dictated by pro-social incentives to lead to emotional arousal if the corresponding decision contradicts the basic instinct of selfish monetary reward maximization. Our results are compatible with this view. First of all, we find a positive and significant correlation (Spearman ρ = 0.12, *p* < 0.01) between arousal and response times. To be more specific, in Figure 4 we plot the distribution of response times in the Inspection treatment per decision type. Recall that in this treatment, the majority choice was the pro-social one, because of the inspection risk. First, in Figure 4A it is seen that officials decide faster when they choose higher quality projects. Similarly, according to Figure 4B, bribing firms take longer to make their decisions. Finally, on Figure 4C we see that losers take less time to decide not to inspect than to inspect. In all these cases, the negative expected profit of the anti-social decision corresponds to higher emotional arousal, as shown by our SCR data. In few words, the more subjects do something against their expected monetary interest, the longer it takes for them to decide, presumably because of conflicting internal motivations.

Thus, in our case, emotional choices can be seen as choices that create conflict or internal dissonance that probably needs even more cognitive processing or reasoning in order to resolve the conflict and make a decision. Rubinstein (2014) associates longer response times with contemplative decisions, as opposed to instinctive and thus faster ones. In Rubinstein (2007), it was argued that instinctive, thus emotional, decisions are usually made faster than those resulting from a cognitively demanding processing. So, in accord with Rubinstein and common sense, more cognitive processing requires more time, although the term instinctive used in Rubinstein (2007) should not be confused with emotional, which, as we show here is more likely in the presence of a moral conflict and specifically, when the decisions made contradict reward maximization.

## 4. Discussion

Economic decisions are often made in contexts generating conflicting motivations. Such conflicts have increasingly attracted the attention of economists, psychologists and decision theorists. Several studies, like Coricelli et al. (2010) and Coricelli et al. (2014) have shown that selfish economic decisions with a negative public externality may cause a moral conflict reflected on the decision-maker's emotional arousal. In fact, both studies had also obtained subjects' self-reported emotions, finding that negative feelings were triggered by non-compliance with a pro-social norm. However, in both of these papers, non-compliance occured in the presence of a threat of punishment through publicity of the photos of subjects engaging in tax evasion. Therefore, any intrinsic aversion to tax evasion or regret for it co-existed with extrinsic threats against anti-social behavior. In this paper, in the context of a public procurement auction with a bribe possibility and punishment options, we find that it is not the violation or compliance with a given ethical norm *per se* which triggers the emotional arousal, but rather the actual decision to act against one's own monetary interest. Complementing our SCR data with response times, we establish that decisions which may or may not be pro-social may cause an increased emotional arousal, as long as they deviate from the objective of monetary reward maximization. From a methodological point of view, our results suggest that, so far, emotional arousal may have been wrongly associated only with unethical behavior, because it may have been triggered by an ethical decision against the decision-maker's selfish motivation. However, emotional arousal is a reliable marker to detect a subject's anxiety due to unethical behavior, while waiting for inspection and punishment by another person.

Future research should pursue obtaining more evidence on the correlation between response times and physiological manifestations of emotions. The extent to which the former can be used as a proxy of the other is of great interest to behavioral economists. Furthermore, more evidence is needed in order to establish the share of *fear of being punished* in the negative emotions associated with the violation of a pro-social norm.

## Author contributions

All authors participated in the design and implementation of the experiments. Pandelis Perakakis designed the process and analyzed the data related to the continuous measurement of skin conductance. Aurora García-Gallego and Nikolaos Georgantzis are responsible for the solution of the theoretical model. Tarek Jaber-López proposed the basic experimental design and performed the analysis of the data, including figures and tables. Nikolaos Georgantzis edited most of the manuscript, helped by all other authors.

### Conflict of interest statement

The authors declare that the research was conducted in the absence of any commercial or financial relationships that could be construed as a potential conflict of interest.

## Footnotes

^1^As for example, in Bolton and Ockenfels (2000).

^2^For example, the studies by Frank and Schulze (2000) and Schulze and Frank (2003) use a single-subject setting to address the issue of compliance to a rule in the context of a student film club in the presence of probabilistic detection. The studies find that economics students are more propense to corruption and that extrinsic motivations work at the cost of crowding out intrinsic moral motivations.

^3^Abbink et al. (2002), Abbink (2004) and Abbink and Hennig-Schmidt (2006) study a two stage-two player game played between a potential briber and a bribee. If a bribe is sent and accepted, respectively, a negative externality is experienced by other subjects in the session. The authors find that less corruption is observed under a strangers matching protocol and that the framing of the situation as bribery matters. The framing matters in the setup adopted by Lambsdorff and Frank (2010, 2011), exposing subjects to an ultimatum game with endogenous framing of first movers' offers as a *bribe* or a *gift*.

^4^Cameron et al. (2009) and Alatas et al. (2009a,b) consider a bribery game between a firm and a public official. Decisions affect the contamination of a river, yielding a negative externality to other players. Culture, gender and experience are shown to matter in this context. Recently, the effects of culture and gender were revisited by Barr and Serra (2009, 2010) in a setup in which the externality affects passive players (citizens). The role of citizens in cultural differences between Pakistan and the US was investigated by Banuri and Eckel (2012) in a setup based on Abbink et al. (2002).

^5^Based on a design by Azfar and Nelson (2007), focusing on the ease of corruption detection and the effect of wages on bribes, Barr et al. (2009) study the role of framing and identities in an experiment run in Ethiopia.

^6^For example, Serra (2012) inverts the usual timing of the underlying game, assuming that the public official is the one who asks for a bribe and the citizen may accept paying it or not.

^7^As opposed to *incidental* emotions, which are not the effect but may cause to some extent the decision. For discussion, see Peters et al. (2006). Examples can be found in Van Dijk and Zeelenberg (2006) and Ariely and Loewenstein (2006).

^8^Sanfey et al. (2003), Bechara (2004), Schwarz (2000) and Naqvi et al. (2006).

^9^Van't Wout et al. (2006), Crone et al. (2004), Bechara and Damasio (2005).

^10^The use of round numbers facilitates subjects' calculations of the consequences of their actions.

^11^The corresponding expected payoff matrix for the firms' bidding subgame is provided in the supplementary material.

^12^These equilibrium bids correspond to the continuous strategy case. With discrete strategies, equilibrium (*Q*_*i*_, *B*_*i*_) = (5, 5) disappears for γ_*i*_ > 0, while as γ_*i*_ increases, equilibria (*Q*_*i*_, *B*_*i*_) = (8, 2), (*Q*_*i*_, *B*_*i*_) = (9, 1) and (*Q*_*i*_, *B*_*i*_) = (10, 0) emerge.

^13^Instructions to the subjects are provided in the supplementary material of the paper.

^14^Specifically, 46 male and 47 female undergraduate student-subjects were recruited by means of the ORSEE software (Greiner, 2004).

^15^7 sessions of 12 subjects and one of 9 subjects.

^16^A photodiode is a type of photodetector capable of converting light changes into electric signals.

^17^Gender differences were statistically non-significant for behavioral results in T1 and physiological responses throughout the study. The only gender differences found in T0 is that women in the role of firms bribe more (Mann-Whitney *z* = −2.07, *p* < 0.05) and in the role of officials exhibit a stronger preference for higher quality bids (Chi-square test *z* = 14.43, *p* < 0.01).
