# Supplementary material for: Physiological and behavioral patterns of corruption
Source: Front Behav Neurosci. 2014 Dec 22;8:434. doi: 10.3389/fnbeh.2014.00434 (PMC4273640; doi:10.3389/fnbeh.2014.00434)
Supplement: Supplementary file 1 [file Presentation1.PDF]

Frontiers in Behavioral Neuroscience

Research Article
29 September 2014

Supplementary Material: Physiological and behavioral patterns of corruption

Tarek Jaber-López [1], Aurora García-Gallego [1,4], Pandelis Perakakis [2] and Nikolaos Georgantzis,[1,3,*]

[1] *Laboratorio de Economía Experimental, Department of Economics, Universitat Jaume I, Castellón, Spain*
[2] *Department of Personality, Evaluation and Psychological Treatment, University of Granada, Spain*
[3] *School of Agriculture Policy and Development, University of Reading, UK*
[4] *Department of Economics, University of Reading, UK*

Correspondence*:
Nikolaos Georgantzis
School of Agriculture Policy and Development, University of Reading, Whiteknights, PO Box 237, RG6 6AR, United Kingdom,
n.georgantzis@reading.ac.uk

1 SUPPLEMENTARY DATA:

1.1 INSTRUCTIONS TO EXPERIMENTAL SUBJECTS (TRANSLATED FROM SPANISH)

Welcome and thanks for your participation in this experiment. Please switch off your mobile phones and secure your belongings away. You are going to participate in an experimental session split into two subsessions of 15 rounds each. You will earn an amount of money which will depend on your decisions and the decisions of other participants in the session. From this moment onwards you must use only the instructions and the computer in front of you. If you have any questions throughout the session, please raise your hand and you will receive an answer by one of the experimentalists. Any communication with other participants will imply your immediate exclusion from the experiment.

1.1.1 [T0] Subsession I: At the beginning of this subsession, you will be assigned one of two roles: 'a firm' or 'an official'. Your role is randomly assigned to you and remains fixed throughout the subsession. You will be anonymously and randomly assigned to a group of three players: two firms and an official. The group will be fixed throughout the subsession. Rounds are independent, in the sense that the payoff consequences of decisions made in any round do not carry over to subsequent rounds. Once this part is finished, the experimentalist will give you new instructions for the second subsession.

- **Decision Making**

In each round, all players receive an endowment of 10 ExCU[1].

[1] Experimental Currency Unit.

If you are a firm: You compete with the other firm of your group for the license of a public project whose quality is beneficial to all players in the group. In each round, you have to post bids on the quality of your project and a monetary transfer which you wish to send privately to the official in your group if you are chosen to undertake the project. Firms' bids are made simultaneously, so that each firm can only know its own bids, but not the bids of the other firm. The quality and the transfer to the official must sum 10, so that if your quality bid is 9 your transfer to the official in case you win will be 1. If you win the auction, apart from your round endowment, you earn a fixed extra profit. In that case, you also have to spend on the transfer to the official double the amount you promised in your bid. If you lose the auction, apart from your initial endowment from each round, your earnings include a profit which is proportional to the quality of the winning project.

If you are an official: In each round, you receive the bids from the firms in your group. Then, you have to choose one of the two projects. Apart from your initial endowment in this round, your earnings include a profit which is proportional to the winner's quality plus the amount, if any, privately transferred to you by the winner.

- **Exact calculation of profits**

From the description of strategies and earnings above, the specific formulas used to calculate your profits in each round (π) are a function of the quality (Q) and transfer (B) bids of the winner, as shown below:

$$\pi_{winner} = 10 + \frac{1}{2} \cdot Q_{winner} + 10 - 2 \cdot B_{winner}$$

$$\pi_{loser} = 10 + \frac{1}{2} \cdot Q_{winner}$$

$$\pi_{official} = 10 + \frac{1}{2} \cdot Q_{winner} + B_{winner}$$

- **Information received**

If you are a firm: At the end of each round, you will receive information on which firm won the license, a reminder of your decisions on quality level and transfer in that round, as well as your profit for that round.

If you are an official: In each round, after firms have made their decisions, their quality and transfer bids will be displayed on your screen before you make a decision. Once you select the winning firm, you will receive information on your profits in this round.

- **Monetary rewards**

In order to determine your payment in this subsession, the computer will randomly choose one of the 15 rounds at the end of the session. The amount of money you will earn from this part of the experiment will be equal to your profits in the randomly chosen round, multiplied by an equivalence ratio of 1 ExCU = $\frac{1}{2}$ Euro.

1.1.2 [T1] Subsession II In this subsession, the context will be exactly the same as in the first one, except for the following:

The roles of subjects and the composition of groups will be re-determined randomly and will remain fixed throughout the remaining rounds of the session.

A new feature is that, in each round, after the winner of the auction has been determined by the official, the loser firm has the option to ask for an inspection of the winning bid. The decision to inspect modifies the subjects' earnings as follows: If a transfer has taken place, the official and the winner of the auction earn nothing in this round. On the contrary, if no transfer is revealed, then, the inspecting loser earns nothing in this round. Finally, if no inspection is activated, players' earnings remain unchanged.

- **Monetary rewards**

In order to determine your payment in this subsession, the computer will randomly choose one of the 15 rounds at the end of the session. The amount of money you will earn from this part of the experiment will be equal to your profits in the randomly chosen round, multiplied by an equivalence ratio of 1 ExCU = $\frac{1}{2}$ Euro.

2 SUPPLEMENTARY TABLES

		Player 2																					
	Bribes	10		9		8		7		6		5		4		3		2		1		0	
player 1	10	5	5	0	10	0	10	0	10	0	10	0	10	0	10	0	10	0	10	0	10	0	10
	9	10	0	6,5	6,5	2,5	10,5	2,5	10,5	2,5	10,5	2,5	10,5	2,5	10,5	2,5	10,5	2,5	10,5	2,5	10,5	2,5	10,5
	8	10	0	10,5	2,5	8	8	5	11	5	11	5	11	5	11	5	11	5	11	5	11	5	11
	7	10	0	10,5	2,5	11	5	9,5	9,5	7,5	11,5	7,5	11,5	7,5	11,5	7,5	11,5	7,5	11,5	7,5	11,5	7,5	11,5
	6	10	0	10,5	2,5	11	5	11,5	7,5	11	11	10	12	10	12	10	12	10	12	10	12	10	12
	5	10	0	10,5	2,5	11	5	11,5	7,5	12	10	12,5	12,5	12,5	12,5	12,5	12,5	12,5	12,5	12,5	12,5	12,5	12,5
	4	10	0	10,5	2,5	11	5	11,5	7,5	12	10	12,5	12,5	14	14	15	13	15	13	15	13	15	13
	3	10	0	10,5	2,5	11	5	11,5	7,5	12	10	12,5	12,5	13	15	15,5	15,5	17,5	13,5	17,5	13,5	17,5	13,5
	2	10	0	10,5	2,5	11	5	11,5	7,5	12	10	12,5	12,5	13	15	13,5	17,5	17	17	20	14	20	14
	1	10	0	10,5	2,5	11	5	11,5	7,5	12	10	12,5	12,5	13	15	13,5	17,5	14	20	18,5	18,5	22,5	14,5
	0	10	0	10,5	2,5	11	5	11,5	7,5	12	10	12,5	12,5	13	15	13,5	17,5	14	20	14,5	22,5	20	20

Table 1Firms' expected payoff matrix for the quality-bribe bidding subgame.
